# *N*,*N*′-Dimethyl­ethylenediammonium dioxalatocuprate(II)

**DOI:** 10.1107/S1600536811025682

**Published:** 2011-07-09

**Authors:** Papa Aly Gaye, Aminata Diassé Sarr, Mohamed Gaye, Morgane Sanselme, Peulon Valérie Agasse

**Affiliations:** a Département de Chimie, Faculté des Sciences et Techniques, Université Cheikh Anta Diop, Dakar, Senegal; b Sciences et Méthodes Séparatives, UPRES EA 3233 IMR, IRCOF, F-76821, Mont-Saint-Aigan, Université de Rouen Cedex, France

## Abstract

The asymmetric unit of the title salt, (C_4_H_14_N_2_)[Cu(C_2_O_4_)_2_], consists of one complex anion and two cationic half-mol­ecules, the other halves being generated by inversion symmetry. The Cu^II^ atom in the anion is coordinated by two bidentate oxalate ligands in a distorted square-planar geometry. Inter­molecular hydrogen bonds, involving the NH groups as donors and O atoms as acceptors, are observed, which lead to the formation of a three-dimensional network structure.

## Related literature

For background to decomposition reactions leading to oxalate anions, see: Kelly *et al.* (2005 ▶); Diallo *et al.* (2008 ▶). For related structures, see: Androš *et al.* (2010 ▶); Fan *et al.* (2001 ▶); Zhang *et al.* (2009 ▶).
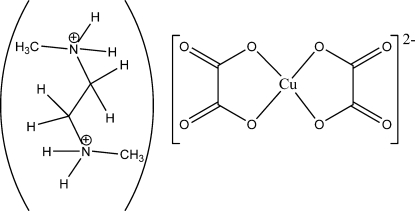


## Experimental

### 

#### Crystal data

(C_4_H_14_N_2_)[Cu(C_2_O_4_)_2_]
*M* *_r_* = 329.75
Triclinic, 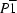

*a* = 5.7734 (5) Å
*b* = 8.4127 (7) Å
*c* = 12.5623 (11) Å
α = 90.443 (1)°
β = 100.715 (1)°
γ = 107.188 (1)°
*V* = 571.46 (8) Å^3^
*Z* = 2
Mo *K*α radiation
μ = 1.95 mm^−1^
*T* = 293 K
0.15 × 0.13 × 0.10 mm

#### Data collection

Bruker SMART CCD diffractometer
4558 measured reflections
2292 independent reflections
2192 reflections with *I* > 2σ(*I*)
*R*_int_ = 0.014

#### Refinement

*R*[*F* ^2^ > 2σ(*F* ^2^)] = 0.024
*wR*(*F* ^2^) = 0.07
*S* = 1.12
2292 reflections
174 parameters
H-atom parameters constrained
Δρ_max_ = 0.35 e Å^−3^
Δρ_min_ = −0.43 e Å^−3^

### 

Data collection: *SMART* (Bruker, 2001 ▶); cell refinement: *SAINT-Plus* (Bruker, 1999 ▶); data reduction: *SAINT-Plus*; program(s) used to solve structure: *SIR92* (Altomare *et al.*, 1993 ▶); program(s) used to refine structure: *SHELXL97* (Sheldrick, 2008 ▶); molecular graphics: *ORTEP-3 for Windows* (Farrugia, 1997 ▶); software used to prepare material for publication: *WinGX* (Farrugia, 1999 ▶).

## Supplementary Material

Crystal structure: contains datablock(s) global, I. DOI: 10.1107/S1600536811025682/wm2502sup1.cif

Structure factors: contains datablock(s) I. DOI: 10.1107/S1600536811025682/wm2502Isup2.hkl

Additional supplementary materials:  crystallographic information; 3D view; checkCIF report

## Figures and Tables

**Table 1:** Selected bond lengths (Å)

Cu1—O1	1.9128 (13)
Cu1—O2	1.9163 (13)
Cu1—O2*A*	1.9184 (13)
Cu1—O1*A*	1.9572 (13)

**Table 2:** Hydrogen-bond geometry (Å, °)

*D*—H⋯*A*	*D*—H	H⋯*A*	*D*⋯*A*	*D*—H⋯*A*
N1*S*—H2*S*1⋯O3*A*^i^	0.90	2.22	2.939 (2)	137
N1*S*—H2*S*2⋯O1*A*^ii^	0.90	2.13	3.018 (2)	169
N2*S*—H4*S*1⋯O4*A*^ii^	0.90	2.15	2.939 (2)	145
N2*S*—H4*S*1⋯O3*A*^ii^	0.90	2.31	3.004 (2)	134
N2*S*—H4*S*2⋯O4^iii^	0.90	2.11	2.861 (2)	140
N2*S*—H4*S*2⋯O3^iv^	0.90	2.58	3.131 (2)	120

Symmetry codes: (i) 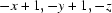; (ii) 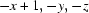; (iii) 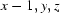; (iv) 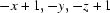.

**Fig. 1.**
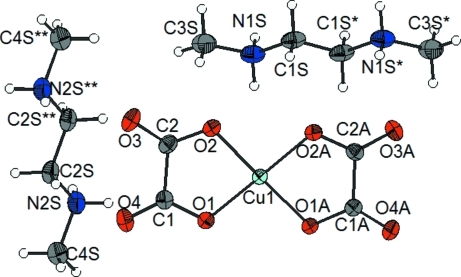
An ORTEP view of the title compound, showing the atom-numbering scheme. Displacement ellipsoids are plotted at the 50% probability level. [Symmetry codes: *) -x + 1, -y + 1, -z; **) -x + 1, -y, -z + 1].

**Fig. 2.**
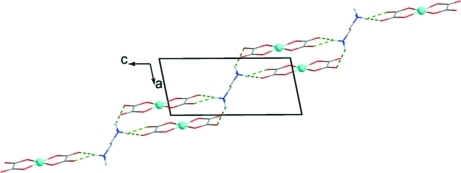
Projection of the structure along the b axis showing the anions connected by organics cations. Broken lines stand for hydrogen bonds.

**Fig. 3.**
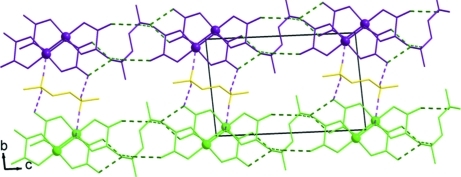
Projection of the structure along the a axis showing the anions connected by organics cations. Broken lines stand for hydrogen bonds.

## supplementary crystallographic information

### Comment

The title salt, (C_4_H_14_N_2_)[Cu(C_2_O_4_)_2_], was obtained as an unexpected product by reaction of the employed ligand (C_6_H_10_N_2_O_2_)_n_, in a methanolic medium. The hydrolytically unstable cyclic ligand apparently is oxidatively hydrolyzed in the presence of metal ions, leading to the oxalate dianion (Diallo *et al.*, 2008; Kelly *et al.*, 2005). This species, which is generated *in situ*, acts with copper(II) ions resulting in the formation of the title compound. A similar reaction was found elsewhere (Zhang *et al.*, 2009).

The structure exhibits an ion-pair complex comprising two cationic half-molecules (the other halves being generated by inversion symmetry) and a [Cu(C_2_O_4_)_2_]^2-^ dianion (Fig. 1).  The Cu^II^ ion is four-coordinated in a slightly distorted square–planar CuO_4_ environment, comprising four O donor atoms from two oxalate ligands [Cu–O, 1.9128 (13), 1.9163 (13), 1.9184 (13) and 1.9572 (13) Å]. The O(1)—Cu—O(2 A) and O(2)—Cu—O(1 A) angles are 179.52 (5) and 178.10 (5)°, respectively, which are slightly smaller than those observed in the complex [K_2_Cu(ox)_2_]^.^4H_2_O (180 °), where ox is oxalate (Fan *et al.*, 2001). The other two oxygen atoms of each oxalate group are not involved in coordination. The two oxalate anions deviate slightly from planarity, with torsion angles of O(1)—C(1)—C(2)—O(2), O(4)—C(1)—C(2)—O(3), O(1A)—C(1A)—C(2A)—O(2A) and O(4A)—C(1A)—C(2A)—O(3A) -2.5 (2), -3.4 (3), 2.8 (2) and 3.8 (2)°, respectively. These values are similar to those found in [Cu(bpy)(C_2_O_4_)(H_2_O)]^.^H_2_C_2_O_4_ (Androš *et al.*, 2010), where bpy is bipyridine.

N—H···O hydrogen-bonding interactions, part of which are bifurcated, between the cations and the complex anions lead to the formation of a three-dimensional network structure (Table 2; Figs. 2, 3).

### Experimental

In a 50 ml round bottom flask dimethyl oxalate (2.36 g, 0.020 mol), dissolved in ethanol (10 ml), was reacted with *N*,*N*'-dimethyl-1,2-diaminoethane (1.77 g, 0.020 mol) in ethanol (10 ml), to yield immediately a quantitative precipitate. The white solid formed was separated by filtration, washed with methanol and ether and dried under vacuum (yield 3.32 g, 58.5%); m.p.= 513 K. ^1^ H NMR in CDCl_3_, δ (p.p.m.): 3.1, s, 12H, –CH3; 3.5, s, 8H, –CH_2_–. ^13^C NMR in CDCl_3_, δ (p.p.m.): 34.86, N—CH_3_, 46.12, N—CH_2_, 157.56, C=O. IR (cm^-1^) 1598 (C=O), 1284 (C—N). Anal. Calc. for C_12_H_20_N_4_O_4_ (%): C, 50.62; H, 7.11; N, 19.68. Found: C, 50.60; H, 7.09; N, 19.71. Mass spectrum (m/*z*) 284, 162, 134, 106, 78. Into a methanolic solution (5 ml) of copper chloride dihydrate (0.2131 g, 1.25 mmol) was added a methanolic solution (10 ml) of the ligand prepared as described above (0.3554 g, 1.25 mmol). The resulting mixture was heated at 333 K for thirty minutes. The green solution was filtered and then allowed to evaporate slowly in open atmosphere. After one week, blue crystals suitable for X-ray analysis were obtained. The crystals were separated, washed with cold methanol and dried (yield: 65%); Anal. Calc. for (C_4_H_14_N_2_)[Cu(C_2_O_4_)_2_](%): C, 29.14; H, 4.28; N, 8.50. Found: C, 29.16; H, 4.26; N, 8.46. Selected IR data (cm^-1^, KBr pellet): 3300, 1637, 1600, 1582, 1197, 764.

### Refinement

All H atoms were located from the Fourier electron density maps, then placed according to their geometrical environment and refined isotropically. They were refined using a riding model with 0.96 Å for methyl H atoms and *U*_iso_(H) = 1.5*U*_eq_(C), with 0.97 Å for methylene H atoms and *U*_iso_(H) = 1.2*U*_eq_(C), and with 0.90 Å for ammonium H atoms and *U*_iso_(H) = 1.2*U*_eq_(N). In addition, a rotating-group model was applied for methyl groups.

### Crystal data

**Table:**

(C_4_H_14_N_2_)[Cu(C_2_O_4_)_2_]	*Z* = 2
*M**_r_* = 329.75	*F*(000) = 338
Triclinic, *P*1	*D*_x_ = 1.916 Mg m^−^^3^
Hall symbol: -P 1	Mo *K*α radiation, λ = 0.71073 Å
*a* = 5.7734 (5) Å	Cell parameters from 2192 reflections
*b* = 8.4127 (7) Å	θ = 1.7–26.4°
*c* = 12.5623 (11) Å	µ = 1.95 mm^−^^1^
α = 90.443 (1)°	*T* = 293 K
β = 100.715 (1)°	Prism, blue
γ = 107.188 (1)°	0.15 × 0.13 × 0.10 mm
*V* = 571.46 (8) Å^3^	

### Data collection

**Table:**

Bruker SMART CCD diffractometer	*R*_int_ = 0.014
graphite	θ_max_ = 26.4°, θ_min_ = 1.7°
ω scans	*h* = −7→7
4558 measured reflections	*k* = −10→10
2292 independent reflections	*l* = −15→15
2192 reflections with *I* > 2σ(*I*)	

### Refinement

**Table:**

Refinement on *F*^2^	Primary atom site location: structure-invariant direct methods
Least-squares matrix: full	Secondary atom site location: difference Fourier map
*R*[*F*^2^ > 2σ(*F*^2^)] = 0.024	Hydrogen site location: inferred from neighbouring sites
*wR*(*F*^2^) = 0.07	H-atom parameters constrained
*S* = 1.12	*w* = 1/[σ^2^(*F*_o_^2^) + (0.0412*P*)^2^ + 0.2106*P*] where *P* = (*F*_o_^2^ + 2*F*_c_^2^)/3
2292 reflections	(Δ/σ)_max_ < 0.001
174 parameters	Δρ_max_ = 0.35 e Å^−^^3^
0 restraints	Δρ_min_ = −0.43 e Å^−^^3^

### Special details

**Table:**

Geometry. All e.s.d.'s (except the e.s.d. in the dihedral angle between two l.s. planes) are estimated using the full covariance matrix. The cell e.s.d.'s are taken into account individually in the estimation of e.s.d.'s in distances, angles and torsion angles; correlations between e.s.d.'s in cell parameters are only used when they are defined by crystal symmetry. An approximate (isotropic) treatment of cell e.s.d.'s is used for estimating e.s.d.'s involving l.s. planes.
Refinement. Refinement of *F*^2^ against ALL reflections. The weighted *R*-factor *wR* and goodness of fit *S* are based on *F*^2^, conventional *R*-factors *R* are based on *F*, with *F* set to zero for negative *F*^2^. The threshold expression of *F*^2^ > σ(*F*^2^) is used only for calculating *R*-factors(gt) *etc*. and is not relevant to the choice of reflections for refinement. *R*-factors based on *F*^2^ are statistically about twice as large as those based on *F*, and *R*- factors based on ALL data will be even larger.

### Fractional atomic coordinates and isotropic or equivalent isotropic displacement parameters (Å^2^)

**Table:**

	*x*	*y*	*z*	*U*_iso_*/*U*_eq_	
Cu1	0.81900 (4)	0.08511 (2)	0.071311 (15)	0.02027 (10)	
O1	0.7688 (2)	−0.09898 (16)	0.16179 (10)	0.0236 (3)	
O2	0.9414 (3)	0.22323 (16)	0.20393 (11)	0.0273 (3)	
O3	0.9874 (3)	0.1983 (2)	0.38225 (12)	0.0400 (4)	
O4	0.8221 (3)	−0.14792 (19)	0.33674 (12)	0.0346 (3)	
O1A	0.7045 (2)	−0.05261 (16)	−0.06558 (10)	0.0244 (3)	
O2A	0.8699 (3)	0.27099 (16)	−0.01838 (11)	0.0274 (3)	
O3A	0.7987 (3)	0.32433 (18)	−0.19228 (12)	0.0325 (3)	
O4A	0.6466 (2)	−0.01666 (17)	−0.24263 (10)	0.0271 (3)	
C1	0.8324 (3)	−0.0538 (2)	0.26338 (15)	0.0230 (4)	
C2	0.9307 (3)	0.1394 (2)	0.28879 (15)	0.0249 (4)	
C1A	0.7074 (3)	0.0373 (2)	−0.14883 (14)	0.0201 (3)	
C2A	0.8000 (3)	0.2289 (2)	−0.11968 (15)	0.0221 (4)	
C1S	0.5705 (4)	0.5131 (2)	0.05810 (18)	0.0323 (4)	
H1S1	0.7161	0.4764	0.0631	0.039*	
H1S2	0.6244	0.6309	0.0809	0.039*	
N1S	0.4111 (3)	0.4180 (2)	0.13069 (15)	0.0328 (4)	
H2S1	0.2785	0.4547	0.1272	0.039*	
H2S2	0.3564	0.3094	0.1074	0.039*	
C2S	0.4947 (4)	−0.0903 (3)	0.50540 (16)	0.0302 (4)	
H3S1	0.6487	−0.1056	0.4941	0.036*	
H3S2	0.4737	−0.1213	0.578	0.036*	
N2S	0.2872 (3)	−0.1980 (2)	0.42503 (13)	0.0329 (4)	
H4S1	0.313	−0.1731	0.3578	0.039*	
H4S2	0.1467	−0.177	0.4324	0.039*	
C3S	0.5449 (4)	0.4354 (3)	0.24519 (18)	0.0394 (5)	
H1S3	0.6805	0.3899	0.25	0.059*	
H1S4	0.434	0.3762	0.2898	0.059*	
H1S5	0.6069	0.5512	0.2698	0.059*	
C4S	0.2574 (5)	−0.3766 (3)	0.4373 (2)	0.0484 (6)	
H2S3	0.4075	−0.3993	0.4303	0.073*	
H2S4	0.124	−0.4417	0.382	0.073*	
H2S5	0.2213	−0.4051	0.5075	0.073*	

### Atomic displacement parameters (Å^2^)

**Table:**

	*U*^11^	*U*^22^	*U*^33^	*U*^12^	*U*^13^	*U*^23^
Cu1	0.02469 (14)	0.01821 (14)	0.01732 (14)	0.00556 (9)	0.00424 (9)	0.00003 (9)
O1	0.0270 (7)	0.0220 (6)	0.0214 (6)	0.0065 (5)	0.0054 (5)	0.0008 (5)
O2	0.0344 (7)	0.0231 (6)	0.0228 (7)	0.0070 (6)	0.0046 (5)	−0.0023 (5)
O3	0.0505 (9)	0.0451 (9)	0.0212 (7)	0.0126 (7)	0.0027 (6)	−0.0065 (6)
O4	0.0398 (8)	0.0394 (8)	0.0268 (7)	0.0139 (7)	0.0089 (6)	0.0120 (6)
O1A	0.0326 (7)	0.0193 (6)	0.0194 (6)	0.0060 (5)	0.0034 (5)	−0.0003 (5)
O2A	0.0389 (8)	0.0198 (6)	0.0229 (7)	0.0069 (6)	0.0075 (6)	0.0005 (5)
O3A	0.0419 (8)	0.0299 (7)	0.0280 (7)	0.0139 (6)	0.0071 (6)	0.0104 (6)
O4A	0.0284 (7)	0.0331 (7)	0.0189 (7)	0.0097 (6)	0.0022 (5)	−0.0022 (5)
C1	0.0189 (8)	0.0285 (9)	0.0243 (9)	0.0101 (7)	0.0063 (7)	0.0032 (7)
C2	0.0221 (9)	0.0290 (9)	0.0236 (9)	0.0092 (7)	0.0024 (7)	−0.0032 (7)
C1A	0.0160 (8)	0.0235 (9)	0.0226 (9)	0.0084 (7)	0.0047 (6)	0.0006 (7)
C2A	0.0212 (8)	0.0224 (8)	0.0254 (9)	0.0095 (7)	0.0063 (7)	0.0033 (7)
C1S	0.0270 (10)	0.0234 (9)	0.0457 (12)	0.0029 (8)	0.0135 (9)	−0.0064 (8)
N1S	0.0272 (8)	0.0244 (8)	0.0462 (10)	0.0054 (7)	0.0098 (7)	−0.0023 (7)
C2S	0.0294 (10)	0.0409 (12)	0.0210 (9)	0.0130 (9)	0.0028 (7)	0.0028 (8)
N2S	0.0340 (9)	0.0379 (9)	0.0244 (8)	0.0084 (7)	0.0039 (7)	0.0050 (7)
C3S	0.0414 (12)	0.0311 (11)	0.0445 (13)	0.0124 (9)	0.0032 (10)	−0.0025 (9)
C4S	0.0696 (17)	0.0385 (12)	0.0354 (12)	0.0118 (12)	0.0131 (11)	0.0060 (10)

### Geometric parameters (Å, °)

**Table:**

Cu1—O1	1.9128 (13)	C1S—H1S2	0.97
Cu1—O2	1.9163 (13)	N1S—C3S	1.485 (3)
Cu1—O2A	1.9184 (13)	N1S—H2S1	0.90
Cu1—O1A	1.9572 (13)	N1S—H2S2	0.90
O1—C1	1.282 (2)	C2S—N2S	1.473 (3)
O2—C2	1.284 (2)	C2S—C2S^ii^	1.511 (4)
O3—C2	1.218 (2)	C2S—H3S1	0.97
O4—C1	1.218 (2)	C2S—H3S2	0.97
O1A—C1A	1.295 (2)	N2S—C4S	1.474 (3)
O2A—C2A	1.275 (2)	N2S—H4S1	0.90
O3A—C2A	1.220 (2)	N2S—H4S2	0.90
O4A—C1A	1.209 (2)	C3S—H1S3	0.96
C1—C2	1.564 (3)	C3S—H1S4	0.96
C1A—C2A	1.558 (2)	C3S—H1S5	0.96
C1S—N1S	1.486 (3)	C4S—H2S3	0.96
C1S—C1S^i^	1.513 (4)	C4S—H2S4	0.96
C1S—H1S1	0.97	C4S—H2S5	0.96
			
O1—Cu1—O2	85.90 (5)	C3S—N1S—H2S1	109.2
O1—Cu1—O2A	179.52 (5)	C1S—N1S—H2S1	109.2
O2—Cu1—O2A	93.63 (6)	C3S—N1S—H2S2	109.2
O1—Cu1—O1A	95.12 (5)	C1S—N1S—H2S2	109.2
O2—Cu1—O1A	178.10 (5)	H2S1—N1S—H2S2	107.9
O2A—Cu1—O1A	85.36 (5)	N2S—C2S—C2S^ii^	110.15 (19)
C1—O1—Cu1	113.02 (11)	N2S—C2S—H3S1	109.6
C2—O2—Cu1	112.98 (12)	C2S^ii^—C2S—H3S1	109.6
C1A—O1A—Cu1	111.82 (11)	N2S—C2S—H3S2	109.6
C2A—O2A—Cu1	113.52 (11)	C2S^ii^—C2S—H3S2	109.6
O4—C1—O1	125.29 (18)	H3S1—C2S—H3S2	108.1
O4—C1—C2	120.57 (17)	C2S—N2S—C4S	112.34 (17)
O1—C1—C2	114.14 (15)	C2S—N2S—H4S1	109.1
O3—C2—O2	125.64 (18)	C4S—N2S—H4S1	109.1
O3—C2—C1	120.45 (17)	C2S—N2S—H4S2	109.1
O2—C2—C1	113.90 (15)	C4S—N2S—H4S2	109.1
O4A—C1A—O1A	125.21 (17)	H4S1—N2S—H4S2	107.9
O4A—C1A—C2A	120.46 (16)	N1S—C3S—H1S3	109.5
O1A—C1A—C2A	114.33 (15)	N1S—C3S—H1S4	109.5
O3A—C2A—O2A	125.78 (17)	H1S3—C3S—H1S4	109.5
O3A—C2A—C1A	119.40 (17)	N1S—C3S—H1S5	109.5
O2A—C2A—C1A	114.82 (15)	H1S3—C3S—H1S5	109.5
N1S—C1S—C1S^i^	110.2 (2)	H1S4—C3S—H1S5	109.5
N1S—C1S—H1S1	109.6	N2S—C4S—H2S3	109.5
C1S^i^—C1S—H1S1	109.6	N2S—C4S—H2S4	109.5
N1S—C1S—H1S2	109.6	H2S3—C4S—H2S4	109.5
C1S^i^—C1S—H1S2	109.6	N2S—C4S—H2S5	109.5
H1S1—C1S—H1S2	108.1	H2S3—C4S—H2S5	109.5
C3S—N1S—C1S	112.06 (16)	H2S4—C4S—H2S5	109.5
			
O2—Cu1—O1—C1	0.30 (12)	O1—C1—C2—O3	176.97 (17)
O1A—Cu1—O1—C1	178.68 (12)	O4—C1—C2—O2	177.15 (17)
O1—Cu1—O2—C2	−1.77 (13)	O1—C1—C2—O2	−2.5 (2)
O2A—Cu1—O2—C2	178.14 (13)	Cu1—O1A—C1A—O4A	179.71 (14)
O1—Cu1—O1A—C1A	178.09 (11)	Cu1—O1A—C1A—C2A	0.11 (17)
O2A—Cu1—O1A—C1A	−1.83 (12)	Cu1—O2A—C2A—O3A	175.09 (15)
O2—Cu1—O2A—C2A	−178.11 (13)	Cu1—O2A—C2A—C1A	−4.23 (19)
O1A—Cu1—O2A—C2A	3.51 (13)	O4A—C1A—C2A—O3A	3.8 (3)
Cu1—O1—C1—O4	−178.63 (15)	O1A—C1A—C2A—O3A	−176.58 (16)
Cu1—O1—C1—C2	0.99 (18)	O4A—C1A—C2A—O2A	−176.83 (16)
Cu1—O2—C2—O3	−176.78 (17)	O1A—C1A—C2A—O2A	2.8 (2)
Cu1—O2—C2—C1	2.64 (19)	C1S^i^—C1S—N1S—C3S	178.0 (2)
O4—C1—C2—O3	−3.4 (3)	C2S^ii^—C2S—N2S—C4S	−175.9 (2)

Symmetry codes: (i) −*x*+1, −*y*+1, −*z*; (ii) −*x*+1, −*y*, −*z*+1.

### Hydrogen-bond geometry (Å, °)

**Table:**

*D*—H···*A*	*D*—H	H···*A*	*D*···*A*	*D*—H···*A*
N1S—H2S1···O3A^i^	0.90	2.22	2.939 (2)	137
N1S—H2S2···O1A^iii^	0.90	2.13	3.018 (2)	169
N2S—H4S1···O4A^iii^	0.90	2.15	2.939 (2)	145
N2S—H4S1···O3A^iii^	0.90	2.31	3.004 (2)	134
N2S—H4S2···O4^iv^	0.90	2.11	2.861 (2)	140
N2S—H4S2···O3^ii^	0.90	2.58	3.131 (2)	120

Symmetry codes: (i) −*x*+1, −*y*+1, −*z*; (iii) −*x*+1, −*y*, −*z*; (iv) *x*−1, *y*, *z*; (ii) −*x*+1, −*y*, −*z*+1.
